# Volatile Metabolites in Liverworts of Ecuador

**DOI:** 10.3390/metabo10030092

**Published:** 2020-03-05

**Authors:** Eduardo Valarezo, Oswaldo Tandazo, Kathia Galán, Jandry Rosales, Ángel Benítez

**Affiliations:** 1 Departamento de Química y Ciencias Exactas, Universidad Técnica Particular de Loja, San Cayetano Alto s/n, 110150 Loja, Ecuador; ortandazo@utpl.edu.ec (O.T.); kcgalan@utpl.edu.ec (K.G.); jandryximena@gmail.com (J.R.); 2 Departamento de Ciencias Biológicas, Universidad Técnica Particular de Loja, San Cayetano Alto s/n, 110150 Loja, Ecuador; arbenitez@utpl.edu.ec

**Keywords:** bryophytes, foliose liverworts, secondary metabolites

## Abstract

Volatile metabolites from *Frullania brasiliensis* Raddi, *Herbertus juniperoideus* (Sw.) Grolle, *Leptoscyphus hexagonus* (Nees) Grolle, and *Syzygiella anomala* (Lindenb. & Gottsche) Steph collected in the south of Ecuador were investigated. Volatile secondary metabolites were extracted by hydrodistillation and analyzed by gas chromatography/flame ionization detector (GC/FID) and Gas chromatography/Mass spectrometry (GC/MS). Sixty-seven volatile compounds were identified in the four species, which represent between 80.12–90.17% of the total chemical composition. The major components were τ-muurolol (32.14%) and Germacrene-D (11.98%) in the essential oil of *F. brasiliensis*, bicyclogermacrene (18.23%), and Caryophyllene oxide (15.29%) in the oil of *H. juniperoideus*, Cabreuva oxide D (33.77%) and Elemol (18.55%) in the oil of *Leptoscyphus hexagonus*, and Silphiperfola-5,7(14)-diene (25.22%) and Caryophyllene oxide (8.98%) in the oil of *Syzygiella anomala*. This is the first report on volatile compounds for the species *Herbertus juniperoideus*, *Leptoscyphus hexagonus*, and *Syzygiella anomala*.

## 1. Introduction

The Ecuador has a very rich liverworts flora over 770 species [1,2,3]. Liverworts produce secondary metabolites in abundance [4] with more than 1500 terpenoids and 350 aromatic compounds flavonoids [5,6,7]. In this context, liverworts produce terpenoids and aromatic compounds, many of which exhibit diverse, interesting biological properties related to antitumor, antimicrobial, antifungal, antioxidative, and insecticidal activities; cytotoxic and insect antifeedant [5]. Several studies have shown abundant secondary metabolites in foliose liverworts. For example, Asakawa et al. [8] studied by gas chromatography coupled to mass spectrometry (GC/MS) the volatile components of 25 taxa of the liverwort family Frullaniaceae from New Zealand, Australia and South America. 

In addition, chemical constituents of *Frullania serrata* were isolated, and their structure was elucidated by nuclear magnetic resonance (1D- and 2D-NMR), high-resolution electrospray ionization mass spectrometry (HR-ESI-MS) and infrared spectroscopy (IR) [9]. The chemical composition of *Frullania tamarisci* essential oil was investigated using gas chromatography coupled to flame ionization detection (GC/FID), GC/MS, and NMR analyses [10]. Compounds of *Frullania hamatiloba* have been isolated and their structures were determined by a combination of the ^1^H- and ^13^C-NMR, MS, ultraviolet-visible spectroscopy (UV), IR, and X-ray crystallographic (X-ray) analysis [11]. In another species of this genus, the *F. brasiliensis* two eremophilanolides were established by NMR and X-ray analysis [12]. 

On the other hand, chemical constituents from *Herbertus subdentatus* (Steph.) Fulford and *H. acanthelius* Spruce have been detected by ^1^H- and ^13^C-NMR, UV, IR, thin-layer chromatography (TLC), and GC/MS [13]. In the family Lophocoleaceae, chemical constituents from *Chiloscyphus pallidus* (Mitt.) Engel & Schuster [14], *Heteroscyphus planus* [15], and *Heteroscyphus billardierei* (Schwägr.) Schiffn. have been isolated and studied by GC/MS and NMR techniques [16]. Finally, secondary metabolites in the liverwort *Syzygiella rubricaulis* (Nees) Stephani has been analyzed by GC/MS [17].

In the Ecuador, liverworts are plants that produce a wide array of biologically active compounds [13], however, less than 3% of these compounds have been investigated. As it is recorded in the bibliography of the large number of existing liverworts species in Ecuador, there is a record of the study of the compounds from *Frullania brasiliensis*, *Herbertus acanthelius*, *Herbertus subdentatus*, *Plagiochila alternans* Lindenb. & Gottsche, *Plagiochila micropterys* Gottsche, *Macrolejeunea pallescens* (Mitt.) Schiffn., *Marchantia plicata* Nees & Mont. [13], *Marchesinia brachiate* (Sw.) Schiffn. [18], *Noteroclada confluens* Taylor, *Symphyogyna brasiliensis* Nees & Mont. [19], and *Syzygiella rubricaulis* (Nees) Stephani [17].

The purpose of the present investigation was the isolation and identification of volatile metabolites of four species of liverworts from Ecuador, three of which, *Herbertus juniperoideus*, *Leptoscyphus hexagonus*, and *Syzygiella anomala*, do not have previous chemical or phytochemical studies.

## 2. Results

### 2.1. Volatile Compounds Isolation

By means of hydrodistillation in a Clevenger-type apparatus, 0.3 mL of essential oil was obtained from 1500 g of *Frullania brasiliensis*, what represents a yield of 0.02% (*v*/*w*). For the species *Herbertus juniperinus*, *Leptoscyphus hexagonus*, and *Syzygiella anomala*, yields close to 0.01% (*v*/*w*) were obtained.

### 2.2. Volatile Compounds Identification

The identification of volatile compounds present in liverworts of Ecuador was carried out by means of gas chromatography equipped with a flame ionization detector (GC/FID) and gas chromatography coupled to a mass spectrometer detector (GC/MS) using capillary nonpolar column DB-5ms. To our knowledge, this is the first report on the chemical composition of volatile compounds from *Herbertus juniperinus*, *Leptoscyphus hexagonus*, and *Syzygiella anomala* (Table 1). In total, sixty-seven chemical constituents were identified in essential oil samples, representing 80.12–90.17% of the total composition. These constituents were grouped into monoterpene hydrocarbons (0.14%), oxygenated monoterpenes (0.79–1.16%), sesquiterpene hydrocarbons (19.61–65.39%), oxygenated sesquiterpenes (23.48–66.24%), diterpene hydrocarbons (0.23–0.87%), and other compounds (0.76–1.16%).

Twenty-seven components were determined in essential oil of *H. juniperinus*, representing 88.21% of the total oil, the principal constituents are found to be sesquiterpene hydrocarbons: bicyclogermacrene (18.23%) and germacrene-D (4.67%) and oxygenated sesquiterpene: caryophyllene oxide (15.29%), spathulenol (11.90%) and viridiflorol (8.93%). Twenty-one components were identified for the essential oil of *L. hexagonus*, representing 85.85% of the total oil, the principal group was oxygenated sesquiterpenes such as cabreuva oxide D (33.77%), Elemol (18.55%) and Viridiflorol (8.03%) and sesquiterpene hydrocarbons like bicyclogermacrene (6.70%). Τ-muurolol (32.14%), germacrene-D (11.98%), Rosifoliol (5.08%), τ-cadinol (4.71%) and elemol (4.19%) were the main compounds of *F. brasiliensis* out of Twenty-five components, representing 80.12% of the total essential oil.

In essential oils of *F. brasiliensis* the principal groups were oxygenated sesquiterpenes (50.81%) and sesquiterpene hydrocarbons (27.47%). Other compounds such as thymol methyl ether, 3-Oxo-7,8-dihydro-β-ionol, and Hexahydrofarnesyl acetone were detected from the essential oils of *H. juniperinus* and *F. brasiliensis*, representing 0.76% and 1.16%, respectively. A monoterpene hydrocarbon β-Phellandrene was detected in essential oils of *S. anomala* and an oxygenated monoterpenes 1-octen-3-ol acetate was detected in the oils of *S. anomala* and *F. brasiliensis*.

## 3. Discussion

Many liverworts are endemic to the southern hemisphere, including Oceania and South America [7]. In the Ecuador (South America), there are around 770 species of liverworts [1,2,3], of which about eleven species have been previously studied. Liverworts (Hepaticae) are a rich source of terpenoids and aromatic compounds [7]. In this research, chemical constituents of studied species of liverworts were mainly grouped into sesquiterpene hydrocarbons (SH) and oxygenated sesquiterpenes (OS). Only one monoterpene hydrocarbon (MH) (β-phellandrene) with RI 1023 was determined; below RI 1000, no compound was identified in any of the four species studied. However, in *Frullania tamarisci* of France [10], three compounds (α-pinene, 1-Octen-3-ol, and β-pinene) with RI below 1000 were identified, which would indicate that it is possible to isolate this type (MH) of compounds from liverworts. 

Around the year 1991, Nagashima et al. [13] investigated terpenoids and aromatic compounds of seven Ecuadorian liverworts. In the spieces *Frullania brasiliensis* found in total two compounds, the sesquiterpenoids Arbusculin B and α-Bisabolol, the relative abundance percentages are not mentioned. In our study, for species *Frullania brasiliensis* twenty-five compounds were identified, the main compound identified for this species was τ-muurolol (32.14%), that like the α-Bisabolol is an oxygenated sesquiterpenes of formula C_15_H_26_O.

Between 2006 and 2001 Bardón et al. [12] studied the nonvolatile compounds from *Frullania brasiliensis* of Argentina, achieving isolate and identify two eremophilanolides, 5-epidilatanolides A and B, as well as a new natural bibenzyl. In this same study, also were identified the nonvolatile compounds eudesmane-type sesquiterpene lactones nepalensolide A, nepalensolide B, (+)-frullanolide, and (+)-dihydrofrullanolide, hopanoid zeorin, four sterols stigmasta-4,22-dien-3,6-dione, stigmasta-4,22-dien-3-one, stigmasterol, and sitosterol, and a trace amount of atraric acid.

The principal constituents in *H. juniperinus* are bicyclogermacrene (18.23%) and caryophyllene oxide (15.29%). For this species, no previous chemical or phytochemical studies have been carried out. However, two Ecuadorian species of the genus *Herbertus*, *H. acanthelius*, and *H. subdentatus*, were previously studied by Nagashima et al. [13] in which identified isocuparene-type sesquiterpenoids as the major components. 

In studies related with Ecuadorian liverwort Nagashima et al. [13,18] report that the compounds 3,4-Dimethoxy-1-vinylbenzene, 2,4,5-trimethoxy-1-vinylbenzene and apigenin-7,4′-dimethylether were identified in *Marchesinia brachiate*. 1,4-Dimethylazulene was isolated from *Plagiochila micropterys* and *Macrolejeunea pallescens*. The major component of *Marchantia plicata* is marchantin A and pinguisane-type sesquiterpenoid is the major component in *Plagiochila alternans*.

In the Ecuadorian species, *Noteroclada confluens* Taylor Ludwiczuk et al. [19] determined that the major component with 49% is an unknown sesquiterpene alcohol (molecular ion M^+^ 222), and that species also produced a large amount of bicyclogermacrene and an unidentified bibenzyl derivative (molecular ion M^+^ 344]. According to Ludwiczuk et al. the main compounds occurring in *Symphyogyna brasiliensis* Nees from Ecuador are Dihydroagarofurane (36.3%) and δ-selinene (20.6%), these species also produced cascarilladiene, selina-4,7-diene, eudesma-5,7(11)-diene, Bicyclogermacrene, thujopsene, calarene, γ- and δ-cuprenene, β-cubebene, bourbon-7(11)-ene and trans-dauca-4(11),7-diene [19]. On the other hand, Costa et al. [17] determined that chemical profiles of lipophilic extracts of *Syzygiella rubricaulis* (Nees) Stephani are rich in sesquiterpenes. Regarding the species *Leptoscyphus hexagonus* and *Syzygiella anomala*, no previous studies have been carried out.

## 4. Materials and Methods

### 4.1. Materials

Dichloromethane and sodium sulfate anhydrous were purchased from Sigma-Aldrich. The standard of aliphatic hydrocarbons was purchased from CHEM SERVICE under the name of Diesel Range Organics Mixture #2-GRO/DRO and with the code M-TPH6X4-1ML. Helium was purchased from INDURA, Ecuador. All chemicals were of analytical grade and used without further purifications.

### 4.2. Plant Material

The plant material of the four species was collected in “El Tiro”, in the province of Loja (Southern Ecuador, latitude, 3°58′59” S; longitude, 79°08’05” W; the altitude ranged from 2800–3000 m a.s.l). The storage and transfer of the plant material were carried out in airtight plastic containers until they are hydrodistilled. The collection temperature was 14–16 °C (ambient temperature), and the transfer temperature was 16–18 °C, the pressure was approximately 80 KPa (ambient pressure). Voucher specimens were deposited in the Herbarium of the Universidad Técnica Particular de Loja (HUTPL)-Bryophytes and lichen collection under the acquisition numbers AB-1299 for *Frullania brasiliensis*, AB-1300 for *Herbertus juniperoideus*, AB-1264 for *Leptoscyphus hexagonus*, AB-1301 for *Syzygiella anomala*. The identity of the plant material was confirmed by the curator of lichens and bryophytes, the mentioned herbarium.

### 4.3. Volatile Compounds Isolation

The volatile compounds isolation was realized from 1500 g of vegetal material of *Frullania brasiliensis*, 4029 g of *Herbertus juniperoideus*, 6045 g of *Leptoscyphus hexagonus* and 4874 g of *Syzygiella anomala*. The material was processed fresh, immediately after arriving at the laboratory, between 8 and 12 h after being collected. The plant material of each species was hydrodistilled for four hours using a Clevenger-type apparatus. Subsequently, each extract sample (essential oil) was dried over sodium sulfate anhydrous and was stored in sealed vials, protecting them from light at 4 °C until being used in the analysis [20].

### 4.4. Gas Chromatography/Flame Ionization Detector (GC/FID)

The analyses of the chemical composition of the essential oils were performed on an Agilent gas chromatograph (model 6890N series) equipped with a flame ionization detector (FID). A nonpolar column DB-5ms (5%-phenyl-methylpolyxilosane) 30 m × 0.25 mm, thickness 0.25 µm was used. An automatic injector (series 7683) in split mode was used. The samples, 1 µL of solution (1/100, *v*/*v*, essential oil/dichloromethane), were injected with a split ratio of 1:50. Helium was used as a carrier gas at 0.9 mL/min in constant flow mode. The initial oven temperature was held at 50 °C for 3 min, and then it was heated to 210 °C with a ramp of 2.5 °C/min, and the temperature was maintained for 3 min until the end. The injector and detector temperatures were of 210 °C and 250 °C, respectively. The retention index (IR) of the compounds was determined based on the standard of aliphatic hydrocarbons, which were injected after the oils at the same conditions.

### 4.5. Gas Chromatography/Mass Spectrometry (GC/MS)

The GC/MS analyses were performed using an Agilent chromatograph coupled to a mass spectrometer (quadrupole) detector (model Agilent series 5973 inert). The spectrometer was operated at 70 eV, electron multiplier 1600 eV, scan rate: 2 scan/s, and mass range: 40–350 *m*/*z*. It was provided with a computerized system MSD-Chemstation D.01.00 SP1. The same columns described in GC/FID section were used. The ion source temperature was set at 250 °C. The identification of the oil components was based on a comparison of both mass spectrum data and relative retention indices with the published literature [21,22,23]. The relative amounts of individual components were calculated based on the GC peak area (FID response) without using a correction factor.

## 5. Conclusions

Volatile metabolites of *Herbertus juniperoideus* (Sw.) Grolle, *Leptoscyphus hexagonus* (Nees) Grolle, and *Syzygiella anomala* (Lindenb. & Gottsche) Steph were qualitatively and quantitatively characterized for the first time, in addition to these three species of liverworts, volatile secondary metabolites of *Frullania brasiliensis* Raddi were studied. A total of sixty-seven compounds were identified, which were mainly grouped into sesquiterpene hydrocarbons and oxygenated sesquiterpenes. The major components were τ-muurolol in *F. brasiliensis*, bicyclogermacrene in *H. juniperoideus*, Cabreuva oxide D in *L. hexagonus* and silphiperfola-5,7(14)-diene in *S. anomala*.

## Figures and Tables

**Table 1:** Volatile metabolites of Frullania brasiliensis (Fb), Herbertus juniperinus (Hj), Leptoscyphus hexagonus (Lh), and Syzygiella anomala (Sa).

Peak N°	Compound ^a,b^	RI	RI ^ref^	Fb	Hj	Lh	Sa	Type	CF	MM (Da)
1	β-Phellandrene	1023	1025	-	-	-	0.14	MH	C_10_H_16_	136.13
2	1-Octen-3-ol, acetate	1107	1110	0.45	0.79	-	1.16	OM	C_10_H_18_O_2_	170.13
3	Thymol methyl ether	1217	1232	1.16	-	-	-	OT	C_11_H_16_O	164.12
4	Bicycloelemene	1321	1331	-	0.33	-	0.23	SH	C_15_H_24_	204.19
5	δ-EIemene	1324	1335	0.10	-	-	-	SH	C_15_H_24_	204.19
6	α-Cubebene	1335	1345	0.42	0.15	-	-	SH	C_15_H_24_	204.19
7	Silphiperfola-5,7(14)-diene	1355	1363	0.25	-	-	25.22	SH	C_15_H_22_	202.17
8	Isoledene	1358	1374	-	-	-	0.10	SH	C_15_H_24_	204.19
9	α-Copaene	1363	1374	0.44	0.27	0.68	-	SH	C_15_H_24_	204.19
10	β-Bourbonene	1370	1387	0.74	0.32	-	-	SH	C_15_H_24_	204.19
11	β-Elemene	1383	1389	3.03	0.78	-	-	SH	C_15_H_24_	204.19
12	Longifolene	1392	1407	1.77	2.58	0.33	-	SH	C_15_H_24_	204.19
13	β-Cubebene	1393	1387	0,63	-	-	-	SH	C_15_H_24_	204.19
14	α-Longipinene	1399	1350	-	-	3.62	-	SH	C_15_H_24_	204.19
15	α-Gurjunene	1412	1409	-	1.15	-	0.73	SH	C_15_H_24_	204.19
16	Caryophyllene	1412	1417	-	-	0.22	0.10	SH	C_15_H_24_	204.19
17	Aromandendrene	1423	1439	1.07	1.61	0.34	-	SH	C_15_H_24_	204.19
18	β-Barbatene	1431	1440	-	-	-	3.99	SH	C_15_H_24_	204.19
19	Aristolediene	1436	1435	-	-	1.49	-	SH	C_15_H_22_	202.17
20	cis-Thujopsadiene	1437	1465	-	-	-	7.00	SH	C_15_H_22_	202.17
21	cis-Thujopsene	1443	1429	-	-	0.18	-	SH	C_15_H_24_	204.19
22	Dehydroaromadendrene	1445	1460	-	-	1.29	1.61	SH	C_15_H_24_	204.19
23	(E)-β-Farnesene	1447	1454	--	3.20	-	1.95	SH	C_15_H_24_	204.19
24	β-Gurjunene	1448	1431	1.54	-	-	-	SH	C_15_H_24_	204.19
25	Germacrene-D	1466	1484	11.98	4.67	-	-	SH	C_15_H_24_	204.19
26	α-Patchoulene	1470	1454	-	-	1.16	-	SH	C_15_H_24_	204.19
27	Eremophilene	1473	1489	-	-	0.39	-	SH	C_15_H_24_	204.19
28	Alloaromadendrene	1474	1458	-	3.30	-	-	SH	C_15_H_24_	204.19
29	Viridiflorene	1476	1496	2.17	3.69	1.33	6.51	SH	C_15_H_24_	204.19
30	Bicyclogermacrene	1480	1500	tr	18.23	6.70	8.42	SH	C_15_H_24_	204.19
31	α-Selinene	1485	1498	-	-	1.30	-	SH	C_15_H_24_	204.19
32	Cuparene	1488	1504	-	-	-	0.55	SH	C_15_H_22_	202.17
33	Cabreuva oxide D	1492	1479	-	-	33.77	-	OS	C_15_H_24_O	220.18
34	Trichodiene	1501	1533	-	-	-	0.18	SH	C_15_H_24_	204.19
35	δ-Cadinene	1505	1522	-	3.38	-	-	SH	C_15_H_24_	204.19
36	Valencene	1508	1496	-	-	0.58	-	SH	C_15_H_24_	204.19
37	Sesquiphellandrene	1513	1521	-	3.05	-	-	SH	C_15_H_24_	204.19
38	trans-Cycloisolongifol-5-ol	1515	1513	-	-	2.57	-	OS	C_15_H_24_O	220.18
39	β-Vetispirene	1517	1493	-	-	-	8.01	SH	C_15_H_22_	202.17
40	α-Calacorene	1527	1544	0.80	-	-	-	SH	C_15_H_20_	200.16
41	γ-Selinene	1528	1522	2.53	-	-	-	SH	C_15_H_24_	204.19
42	γ-Dehydro-Ar-himachalene	1545	1530	-	-	-	0.79	SH	C_15_H_20_	200.16
43	Maaliol	1553	1566	-	-	-	0.84	OS	C_15_H_26_O	222.20
44	Caryophyllene oxide	1562	1582	-	15.29	-	8.98	OS	C_15_H_24_O	220.18
45	Spathulenol	1562	1577	-	11.90	0.57	-	OS	C_15_H_24_O	220.18
46	β-Oplopenone	1564	1575	-	-	-	6.4	OS	C_15_H_24_O	220.18
47	Elemol	1570	1548	4.19	-	18.55	-	OS	C_15_H_26_O	222.20
48	Globulol	1571	1590	-	-	-	1.83	OS	C_15_H_26_O	222.20
49	Viridiflorol	1579	1592	3.17	8.93	8.03	1.01	OS	C_15_H_26_O	222.20
50	Cubeban-11-ol	1582	1595	-	-	-	0.38	OS	C_15_H_26_O	222.20
51	Ledol	1591	1602	-	-	2.57	-	OS	C_15_H_26_O	222.20
52	Rosifoliol	1592	1600	5.08	-	-	1.04	OS	C_15_H_26_O	222.20
53	Muurola-4,10(14)-dien-1β-ol	1613	1630	-	-	-	0.36	OS	C_15_H_24_O	220.18
54	τ-muurolol	1642	1640	32.14	2.14	-	-	OS	C_15_H_26_O	222.20
55	τ-cadinol	1648	1638	4.71	-	-	-	OS	C_15_H_26_O	222.20
56	Valerenal	1650	1668	-	-	-	0.18	OS	C_15_H_22_O	218.17
57	Torreyol	1657	1656	0.66	-	-	-	OS	C_15_H_26_O	222.20
58	Selin-11-en-4-α-ol	1667	1658		0.82	-	-	OS	C_15_H_26_O	222.20
59	Acorenone	1673	1692	0.86	-	-	-	OS	C_15_H_24_O	220.18
60	3-Oxo-7,8-dihydro-β-ionol	1711	1695	-	0.48	-	-	OT	C_13_H_20_O_2_	208.15
61	Drimenol	1752	1757	-	-	0.18	-	OS	C_15_H_26_O	222.20
62	Aristolone	1757	1762	-	-	-	2.46	OS	C_15_H_22_O	218.17
63	Hexahydrofarnesyl acetone	1833	1843	-	0.28	-	--	OT	C_18_H_36_O	268.28
64	5,15-Rosadiene	1904	1896	-	0.39	-	-	DH	C_20_H_32_	272.25
65	Sandaracopimaradiene	1935	1935	0.23	0.17	-	-	DH	C_20_H_32_	272.25
66	Sclarene	1985	1974	-	0.16	-	-	DH	C_20_H_32_	272.25
67	Kaurene	2060	2042	-	0.15	-	-	DH	C_20_H_32_	272.25
Monoterpene hydrocarbons (MH)				-	-	-	0.14			
Oxygenated monoterpenes (OM)				0.45	0.79	-	1.16			
Sesquiterpene hydrocarbons (SH)				27.47	46.71	19.61	65.39			
Oxygenated sesquiterpenes (OS)				50.81	39.08	66.24	23.48			
Diterpene hydrocarbons (DH)				0.23	0.87	-	-			
Other compounds (OT)				1.16	0.76	-				
Total identified				80.12	88.21	85.85	90.17			

^a^ Compounds ordered according to the elution order in the column DB5-Ms. ^b^ All compounds were identified by MS and RI: MS, by comparison of the mass spectrum with those of the computer mass libraries Wiley 7, Adams 21 and NIST 05 22; RI, by comparison of RI with those reported in the literature; 21, 22, and 23. tr, trace (<0.05%); -, not detected; RI, retention indices in the a-polar column (DB5-MS); RI^ref^, references: 21, 22, and 23. CF, Chemical Formula; MM, Monoisotopic mass.
